# Early Therapeutic Response Predicts Outcome in Chronic Constipation: A Multicenter Prospective Observational Study

**DOI:** 10.14740/gr2071

**Published:** 2026-01-04

**Authors:** Tadayuki Oshima, Seiji Futagami, Yoshimasa Tanaka, Mariko Hojo, Kimio Isshi, Kazuki Kakimoto, Yujiro Uchiyama, Hiroshi Iida, Atsushi Oshio, Koji Nakada

**Affiliations:** a Department of Gastroenterology and Hepatology, Hyogo Medical University, Nishinomiya, Japan; b Department of Gastroenterology, Okazaki City Medical Association Public Health Center, Okazaki, Japan; c Division of Gastroenterology, Nippon Medical School, Tokyo 113-0001, Japan; d Department of Medicine and Bioregulatory Science, Graduate School of Medical Sciences, Kyushu University, Fukuoka, Japan; e Department of Gastroenterology, Juntendo University Faculty of Medicine, Tokyo, Japan; f Isshi Gastrointestinal Clinic, Tokyo, Japan; g 2nd Department of Internal Medicine, Osaka Medical and Pharmaceutical University, Osaka, Japan; h Uchiyama Icho-ka Clinic, Tokyo, Japan; i Department of Hepatology and Gastroenterology, Yokohama City University Hospital, Yokohama, Japan; j Faculty of Letters, Arts and Sciences, Waseda University, Tokyo, Japan; k Department of Laboratory Medicine, The Jikei University Daisan Hospital, Tokyo, Japan; l The Chronic Constipation-Therapeutic Efficacy and Satisfaction Test (CC-TEST) Study Group

**Keywords:** Chronic constipation, Patient’s impression, Patient-reported outcome, Predictive accuracy, Spontaneous bowel movement

## Abstract

**Background:**

Chronic constipation, common in clinical practice, requires treatment to enhance quality of life and possibly extend life expectancy. However, predictors of treatment efficacy remain largely unexplored. This study aimed to identify factors predicting treatment success in patients with chronic constipation.

**Methods:**

A multicenter, prospective observational study evaluated patients with moderate to severe chronic constipation using the Chronic Constipation-Therapeutic Efficacy and Satisfaction Test (CC-TEST) questionnaire. Symptoms were assessed before treatment and at 2 and 4 weeks post-treatment. Multivariate analyses identified predictive factors based on three treatment efficacy assessment criteria: patient’s impression, numeric rating scale (NRS) for symptom intensity, and spontaneous bowel movement (SBM) frequency status.

**Results:**

Constipation medications were administered to 97 patients, with significant symptom improvements observed at 2 and 4 weeks (CC-TEST). The greatest effects were seen in hard stools, difficulty in defecation, and infrequent bowel movements. In the multiple regression analysis, baseline clinical characteristics and symptom profiles were not significant predictors of treatment response. Incorporating 2-week treatment responsiveness revealed that non-responsiveness at 2 weeks (β = 0.487), and a lower stool symptom subscale score (β = -0.344), were associated with poorer patient’s impression. For the NRS, non-responsiveness at 2 weeks (β = 0.279) was a significant predictor. For SBM, non-responsiveness at 2 weeks (β = -0.274) predicted outcomes. Including 2-week non-responsiveness improved the predictive accuracy for 4-week efficacy.

**Conclusions:**

The therapeutic response at 2 weeks is the most significant predictor of subsequent treatment response at 4 weeks in patients with chronic constipation.

## Introduction

Constipation is a common condition, affecting approximately 7% to 12% of the population according to the Rome IV criteria [1, 2]. Chronic constipation significantly impairs quality of life (QOL) [3], and effective treatment strategies are crucial due to its association with increased mortality [4, 5].

Current guidelines recommend initial treatment with lifestyle modifications and osmotic laxatives, such as magnesium salts and polyethylene glycol (PEG), as first-line therapies [6, 7]. While double-blind, placebo-controlled trials typically assess therapeutic effects over 4 weeks [8], the optimal duration for assessing patient satisfaction remains unclear. Extended evaluation periods may expose patients to prolonged symptoms when treatment is ineffective. Therefore, predicting treatment non-responsiveness in clinical practice is essential for timely treatment intensification or modification to improve patient satisfaction.

However, clinical factors predicting treatment non-responsiveness in constipation remain poorly defined. Although stool form and bowel movement frequency have been suggested as potential predictors [7, 9], their predictive accuracy remains limited. This highlights the need to explore alternative predictive factors.

We hypothesize that early treatment response may predict subsequent therapeutic outcomes in patients with constipation. This study assessed treatment efficacy at 2 and 4 weeks and conducted multivariate analyses to identify predictors of non-responsiveness, including both clinical factors and early treatment response, to improve predictive accuracy.

## Materials and Methods

### Study design

**Figure 1:**
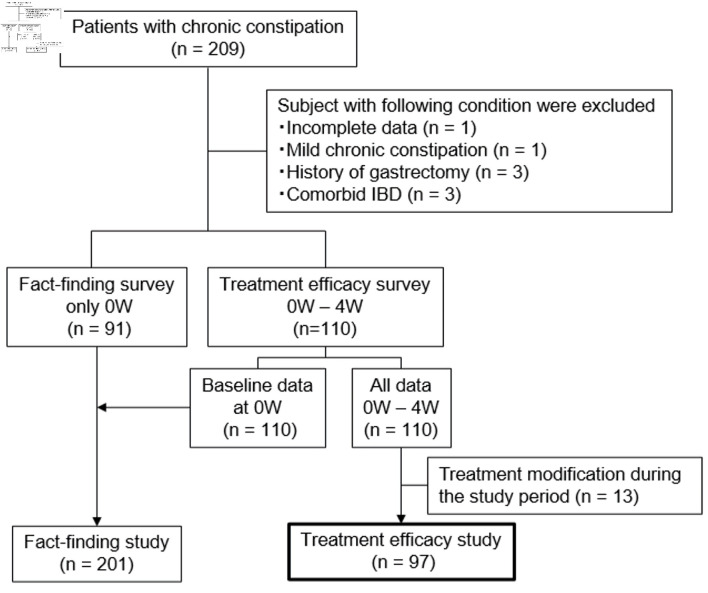
Flowchart of study participants. W: week; IBD: inflammatory bowel disease.

This multicenter prospective observational study was conducted at 17 institutions in Japan from May 7, 2019, to February 27, 2022. The study adhered to the principles outlined in the Declaration of Helsinki (2013) and received ethical approval from the Jikei University Ethical Committee in Tokyo (approval number 30-465(9486)) or from each participating institution. It was registered with the University Hospital Medical Information Network Center Clinical Trials Registry in Japan (reference number UMIN000035794). The overall project comprised two studies: a fact-finding study [10] and a treatment efficacy study. The present report focuses exclusively on the analyses of the treatment efficacy study (Fig. 1). The primary outcome of this study was to identify clinical and early-treatment factors predicting therapeutic response at 4 weeks.

### Patients

Outpatients with moderate or severe chronic constipation, where symptoms significantly impacted daily life and required new or more intensive treatment, were recruited. The planned target sample size for both studies combined was 800 patients, determined based on feasibility considerations across the participating centers. However, patient recruitment was substantially reduced during the coronavirus disease 2019 (COVID-19) pandemic, resulting in a final analyzed sample of 97 patients for this treatment efficacy study. Patients in the treatment efficacy study received the most appropriate constipation medication from attending physicians. All questionnaire data were anonymized before analysis, and the assessors evaluating the responses were blinded to the patients’ treatment details and clinical background information. Inclusion criteria were as follows: 1) moderate or severe chronic constipation, operationally defined as constipation that compromises daily functioning and warrants initiation or escalation of therapy based on clinical judgment by the attending physician; 2) age 20 years or older, of either sex; 3) provision of voluntary consent; and 4) ability to comprehend the questionnaire. Exclusion criteria were as follows: 1) presence of alarm features like vomiting, gastrointestinal tract bleeding (including hematemesis, bloody stool, anemia, etc.); 2) history of gastrointestinal tract resection or vagotomy; 3) diseases or prior surgeries potentially influencing questionnaire outcomes more than constipation; 4) organ failure or mental illness; 5) confirmed or suspected malignancy; 6) pregnancy, potential pregnancy, or breastfeeding; 7) unsuitability for the study per responsible physician; and 8) patients who underwent treatment modification during the study period were excluded from the efficacy analysis.

### Assessments

Patient characteristics were recorded using a comprehensive questionnaire encompassing age, sex, height, weight, constipation duration, impact on daily activities, constipation management strategies (dietary and beverage modifications, supplements, over-the-counter medications (OTCMs)), prescribed medications for constipation, history of abdominal surgeries (laparotomy/laparoscopy), childbirth frequency (vaginal/cesarean section), chronic constipation-related comorbidities and constipation-inducing pharmacological agents. Evaluation used the Chronic Constipation-Therapeutic Efficacy and Satisfaction Test (CC-TEST) [10] (Table 1) at baseline, at 2 weeks, and 4 weeks post-therapy. All questionnaires were completed and sent to the data center by participants.

**Table 1:** Structure of CC-TEST

Domain	Subdomain	Item number	Items	Response form	Subscale	Definitions of SS
Symptoms	Symptoms	Item 1 - 7				
		Item 1	Infrequent bowel movements	7-point LS	Stool Sx-SS	Mean of items 1and 2
		Item 2	Hard stool		Defecation Sx-SS	Mean of items 3, 4 and 7
		Item 3	Difficulty in defecation		Abdominal Sx-SS	Mean of items 5 and 6
		Item 4	Feeling of incomplete bowel movements		Total symptom score	Mean of stool Sx-SS, defecation Sx-SS and abdominal Sx-SS
		Item 5	Abdominal pain			
		Item 6	Abdominal discomfort			
		Item 7	Anal pain during defecation			
	Defecation status	Item 8 - 12				
		Item 8	Number of bowel movements during the past week	Number		
		Item 9	Number of forced bowel movements during the past week			
			Number of spontaneous bowel movements during the past week (item 8 - item 9)			
		Item 10	Bristol Stool Form Scale	7-point LS		
		Item 11	Time to defecate	6-point LS		
		Item 12	Defecation assistance	5-point LS		
Impact for daily life		Item 13 - 16				
		Item 13	Dissatisfaction with daily activity	5-point LS	Dissatisfaction for daily life-SS	Mean of items 13, 14, 15 and 16
		Item 14	Dissatisfaction with the mood			
		Item 15	Dissatisfaction with eating			
		Item 16	Dissatisfaction with overall constipation symptoms			
Therapeutic response		Item 17 - 20				
		Item 17	Desire for additional medication	5-point LS		
		Item 18	Patient’s impression of the therapy			
		Item 19	Numeric rating scale	0 - 10 scale		
		Item 20	Compliance with the medication	5-point LS		

CC-TEST: Chronic Constipation-Therapeutic Efficacy and Satisfaction Test; LS: Likert scale; Sx: symptom; SS: subscale.

### Evaluation of the chronic constipation symptoms

The stool symptoms (Sx) subscale (SS) averaged infrequent bowel movements (Q1) and hard stool (Q2). The defecation Sx-SS averaged difficulty in defecation (Q3), feeling of incomplete bowel movements (Q4), and anal pain during defecation (Q7). The abdominal Sx-SS averaged abdominal pain (Q5) and abdominal discomfort (Q6). The total symptom score averaged stool Sx-SS, defecation Sx-SS, and abdominal Sx-SS. Dissatisfaction for daily life (SS) averaged dissatisfaction for daily activities (Q13), mood (Q14), eating (Q15), and constipation (symptoms and defecation status in general) (Q16).

### Outcome measures

To assess chronic constipation treatment response, three outcome measures were used: 1) patient’s impression of therapy, scored on CC-TEST Q18 (1 = extremely improved, 2 = improved, 3 = slightly improved, 4 = no change, 5 = aggravated); 2) relative symptom intensity by an 11-point NRS (0 = no symptoms to 10 = symptoms before medication); and 3) spontaneous bowel movements (SBMs) per week.

### Definition of responder

Responders were defined based on: 1) patient’s impression: improved or better rating; 2) NRS: score of ≤ 5; and 3) SBM: ≥ 3 per week, with ≥ 1 increase from baseline [10]. The responders/non-responders ratios, residual symptoms at 2 and 4 weeks, and their significance during early (2 weeks) versus subsequent stages (4 weeks) were analyzed. To predict therapeutic responsiveness at 4 weeks, we examined whether adding the treatment response at 2 weeks (responder/non-responder) to the clinical factors (sex, age, body mass index (BMI), duration of constipation, stool Sx-SS, defecation Sx-SS, abdominal Sx-SS, weekly SBM frequency, and Bristol Stool Form Scale (BSFS)) [11] would enhance the predictive power in multiple regression analysis. To quantify deviations from normal stool form and bowel movement frequency, we calculated the absolute differences from BSFS score of 4 (|BSFS - 4|) and from seven spontaneous bowel movements per week (|SBM - 7|), respectively.

### Statistical analysis

Responder/non-responder ratios at 2 and 4 weeks were compared using Chi-squared tests. Multiple regression analyses identified factors influencing therapeutic response: sex, age, BMI, constipation duration, stool Sx-SS, defecation Sx-SS, abdominal Sx-SS, SBMs per week, and BSFS. Early therapeutic response at 2 weeks (responder/non-responder) was included as an explanatory variable, with 4-week outcomes (patient’s impression, NRS, and SBM frequency) as the objective variable. JMP 12.0.1 software (SAS Institute Inc., Cary, NC, USA) conducted data analysis with a significance level of 0.05. Effect sizes were interpreted by Cohen’s guidelines; small, medium, and large (β ≥ 0.10, 0.30, 0.50, R^2^ ≥ 0.02, 0.13, and 0.26) [12].

## Results

### Patient characteristics

Among the 110 enrolled patients, 13 were excluded from the efficacy analysis because their treatment was modified during the study period owing to insufficient therapeutic response in 11 patients and dose reduction for diarrhea in two patients (Fig. 1). The remaining 97 patients were included in the final analysis. Demographic data are presented in Table 2. Of these, 69 patients (71%) were female, with a mean age of 66.4 ± 15.4 years and a mean BMI of 21.7 ± 3.2 kg/m^2^. The mean duration of symptom was 165.6 ± 169.7 months. Forty patients (41%) had a history of abdominal surgery, including appendectomy (16 cases), gynecological surgery (12 cases), and cholecystectomy (seven cases). OTCM and prescribed medications were used by 22 (23%) and 70 (72%) patients, respectively, prior to the study. The medications prescribed in the treatment efficacy study were varied and not standardized (Supplementary Material 1, gr.elmerpub.com). Coexisting disorders were present in 71 (73%) patients, and medications known to cause constipation were used by 57 (60%) patients.

**Table 2:** Patient Characteristics

Age (mean ± SD)	66.4 ± 15.4
Sex	
Female	69 (71%)
Male	28 (29%)
BMI (mean ± SD)	21.7 ± 3.2
Duration of constipation, month (mean ± SD) (month)	165.6 ± 169.7
History of hospital visit due to constipation, yes	69 (71%)
Severity of constipation	
Severe	8 (8%)
Moderate	89 (92%)
OTCM, yes	22 (23%)
Pre-study medication, yes	70 (72%)
Magnesium oxide	33
New constipation treatment medication	26
Stimulant laxatives	18
Kampo medicine	13
Others	21
Number of pre-study medication	
1	36
2	23
3	6
4	2
5	2
Abdominal surgery, yes	40 (41%)
Open	28
Laparoscopic surgery	8
Unclear	4
Childbirth, yes	42 (61%)
Vaginal delivery	35
Cesarean section	6
Unclear	1
Coexisting disorders, yes	71 (73%)
Endocrine and metabolic disorders	35
Neurological disorders	8
Collagen disease	5
Degenerative disorders	1
Mental disorders	13
Colorectal and anal disorders	33
Others	11
Number of coexisting conditions	
1	44
2	20
3	6
4	1
Medications causing constipation, yes	57 (60%)
Number of medications causing constipation	
1	29
2	22
3	6
Types of study prescription medications	
New constipation treatment medication	87
Stimulant laxatives	20
Magnesium oxide	23
Kampo medicine	7
Others	25
Number of study medication	
1	55
2	22
3	14
4	3
5	3

BMI: body mass index; OTCM: over-the-counter medication; SD: standard deviation.

### Therapeutic effectiveness in chronic constipation patients after 4 weeks of treatment

At 2 and 4 weeks post-treatment, CC-TEST domains (symptoms, defecation status, and daily life impact) significantly improved, with no increase in forced bowel movements in the previous week (Table 3). Comparative analysis of CC-TEST items/subscales indicated greater efficacy at 4 weeks versus 2 weeks, suggesting time-dependent treatment effectiveness. Among evaluated constipation symptoms, the greatest treatment efficacy was observed for hard stools, difficulty in defecation, and infrequent bowel movements.

**Table 3:** Effectiveness of Treatment

Items/subscales	Baseline (n = 109)		2 W (n = 109)		0 W vs. 2 W			4 W (n = 107)		0 W vs. 4 W		
	Mean	SD	Mean	SD	Δscore (2 W - 0 W)	P value	Cohen’s *d*	Mean	SD	Δscore (4 W - 0 W)	P value	Cohen’s *d*
Infrequent bowel movements	4.1	1.3	3.1	1.4	-1.0	< 0.001	0.73	2.8	1.4	-1.3	< 0.001	0.94
Hard stool	4.4	1.5	3.0	1.6	-1.4	< 0.001	0.89	2.7	1.7	-1.7	< 0.001	1.07
Difficulty in defecation	4.5	1.5	3.1	1.6	-1.5	< 0.001	0.95	2.9	1.7	-1.6	< 0.001	1.00
Feeling of incomplete bowel movements	3.8	1.4	3.2	1.5	-0.6	< 0.001	0.43	3.1	1.5	-0.7	< 0.001	0.47
Abdominal pain	2.9	1.7	2.3	1.2	-0.6	0.001	0.39	2.3	1.3	-0.6	< 0.001	0.38
Abdominal discomfort	4.1	1.5	3.2	1.5	-0.8	< 0.001	0.54	3.1	1.5	-0.9	< 0.001	0.63
Anal pain during defecation	2.9	1.7	2.3	1.4	-0.6	0.002	0.42	2.2	1.3	-0.8	< 0.001	0.51
Stool Sx-SS	4.3	1.3	3.1	1.4	-1.2	< 0.001	0.89	2.8	1.4	-1.5	< 0.001	1.12
Defecation Sx-SS	3.8	1.2	2.8	1.2	-0.9	< 0.001	0.75	2.7	1.3	-1.0	< 0.001	0.81
Abdominal Sx-SS	3.5	1.4	2.8	1.2	-0.7	< 0.001	0.54	2.7	1.2	-0.8	< 0.001	0.57
Total symptom score	3.8	1.1	2.9	1.1	-0.9	< 0.001	0.84	2.7	1.2	-1.1	< 0.001	0.95
Number of bowel movements during the past week	4.4	4.7	6.9	10.5	2.5	0.020	0.30	6.1	5.1	1.7	0.001	0.34
Number of forced bowel movements during the past week	2.2	3.8	3.0	5.2	0.8	0.102	0.17	2.9	4.6	0.7	0.133	0.16
Number of spontaneous bowel movements during the past week	2.2	3.9	3.9	7.2	1.5	0.016	0.29	3.2	4.0	1.0	0.012	0.25
|SBM-7|	5.6	2.6	5.6	5.4	0.1	0.916	0.01	4.7	2.9	-0.9	0.012	0.31
Bristol Stool Form Scale	2.7	1.8	4.0	1.7	1.3	< 0.001	0.73	4.1	1.7	1.3	< 0.001	0.75
|BSFS-4|	1.9	1.1	1.4	1.0	-0.5	< 0.001	0.48	1.3	1.1	-0.6	< 0.001	0.52
Time to defecate	2.6	1.3	2.0	1.1	-0.6	< 0.001	0.45	1.9	1.1	-0.7	< 0.001	0.56
Defecation assistance	2.3	1.2	1.7	1.1	-0.6	< 0.001	0.48	1.6	1.0	-0.7	< 0.001	0.60
Dissatisfaction with daily activity	2.6	1.1	2.4	1.2	-0.2	0.034	0.19	2.2	1.1	-0.4	< 0.001	0.33
Dissatisfaction with the mood	2.8	1.2	2.4	1.2	-0.3	0.004	0.27	2.3	1.1	-0.5	0.004	0.40
Dissatisfaction with eating	2.3	1.3	1.9	1.2	-0.4	0.001	0.35	1.9	1.1	-0.4	< 0.001	0.34
Dissatisfaction with overall constipation symptoms	3.6	1.1	2.9	1.1	-0.7	< 0.001	0.61	2.7	1.3	-0.8	< 0.001	0.71
Dissatisfaction with daily life-SS	2.8	0.9	2.4	1.0	-0.4	< 0.001	0.46	2.3	0.9	-0.5	< 0.001	0.58

Interpretation of effect size (Cohen’s *d*): small ≥ 0.20, medium ≥ 0.50, large ≥ 0.80. W: week; SD: standard deviation; Sx: symptom; SS: subscale; BSFS: Bristol Stool Form Scale; SBM: spontaneous bowel movement.

### Factors influencing therapeutic response after 4 weeks of treatment

**Figure 2:**
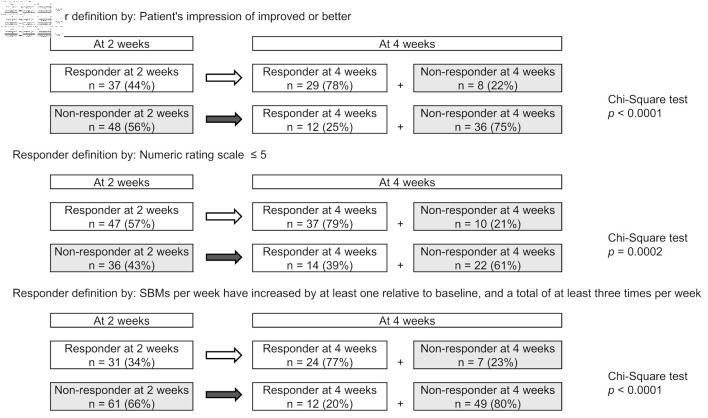
Correlation between treatment reactivity at 2 and 4 weeks of treatments. (a) Responder defined by patient’s impression of improved or better (Q18). (b) Responder defined by numeric rating scale ≤ 5 (Q19). (c) Responder defined by spontaneous bowel movement (SBM) responder.

Among 2-week responders, 77-79% remained as responders at 4 weeks; among non-responders, 20-39% became responders, with 61-80% remaining non-responders (Fig. 2). Significant associations were found between 2-week responders remaining responders and non-responder remaining non-responders in all responder definitions (P < 0.001).

To examine the influence of pretreatment clinical characteristics and symptom profiles on the 4-week therapeutic response, multiple regression analyses were conducted (Table 4). The objective variables included the patient’s impression of therapy, NRS relative symptom intensity, and the frequency of SBM in the week prior to treatment week 4. Multiple regression analysis demonstrated that baseline clinical characteristics and symptom profiles were not significant predictors of treatment response, as indicated by non-significant model fits (R^2^, P > 0.05) across patient’s impression, NRS, and SBM frequency.

**Table 4:** Clinical Factors Associated With Therapeutic Response at 4 Weeks (Multiple Regression Analysis)

Factor	Patient’s impression		NRS		SBM frequency	
	β	P value	β	P value	β	P value
Age	-0.292	0.022	0.037	0.778	0.069	0.577
Sex (female)	-0.117	0.368	-0.001	0.997	0.047	0.713
BMI	-0.040	0.717	0.106	0.364	0.003	0.980
Duration of constipation	-0.009	0.938	-0.023	0.848	0.168	0.145
Stool Sx-SS	-0.263	0.125	-0.214	0.217	-0.149	0.371
Defecation Sx-SS	0.028	0.875	0.008	0.963	-0.022	0.900
Abdominal Sx-SS	-0.011	0.937	0.089	0.557	-0.027	0.854
|SBM-7|	0.158	0.170	0.268	0.029	0.116	0.313
|BSFS-4|	-0.085	0.472	-0.002	0.987	0.133	0.251
R^2^	0.146	0.186	0.118	0.385	0.086	0.580
Interpretation (effect size)	β	R^2^				
Small	≥ 0.1	≥ 0.02				
Medium	≥ 0.3	≥ 0.13				
Large	≥ 0.5	≥ 0.26				

BSFS: Bristol Stool Form Scale; BMI: body mass index; NRS: numerical rating scale; SBM: spontaneous bowel movement; Sx: symptom; SS: subscale.

In addition, multiple regression analyses were performed by incorporating the 2-week treatment effect (responder/non-responder) into the pretreatment clinical factors as explanatory variables (Table 5). For the patient’s impression, non-responder status at 2 weeks (β = 0.558, P < 0.001), younger age (β = -0.271, P = 0.016), and lower stool Sx-SS (β = -0.344, P < 0.024) significantly correlated with a poorer patient’s impression at 4 weeks. For NRS, non-responder status at 2 weeks (β = 0.429, P = 0.000) correlated with a larger NRS score at 4 weeks. Regarding SBM frequency, non-responder status at 2 weeks (β = -0.451, P < 0.001) predicted fewer SBMs at 4 weeks. The coefficients of determination (R^2^ = 0.392, 0.279, and 0.274 for patient’s impression, NRS, and SBM frequency, respectively) indicated that including the 2-week therapeutic response substantially improved predictive power.

**Table 5:** Effect of Adding Non-Response at Week 2 on the Prediction of Therapeutic Response at 4 Weeks (Multiple Regression Analysis)

Factor	Patient’s impression		NRS		SBM frequency	
	β	P value	β	P value	β	P value
Age	-0.271	0.016	-0.018	0.882	0.083	0.460
Sex (female)	-0.138	0.228	0.020	0.876	0.001	0.996
BMI	-0.136	0.174	0.043	0.695	0.062	0.537
Duration of constipation	0.073	0.464	0.040	0.715	0.142	0.173
Stool Sx-SS	-0.344	0.024	-0.315	0.053	-0.072	0.638
Defecation Sx-SS	0.064	0.680	0.007	0.965	-0.093	0.572
Abdominal Sx-SS	0.030	0.815	0.085	0.549	0.026	0.844
|SBM-7|	-0.039	0.716	0.217	0.06	0.165	0.115
|BSFS-4|	-0.015	0.885	-0.010	0.927	0.098	0.349
Non-responder at week 2	0.558	< 0.001	0.429	< 0.001	-0.451	< 0.001
R^2^	0.392	< 0.0001	0.279	0.009	0.274	0.003
Interpretation (effect size)	β	R^2^				
Small	≥ 0.1	≥ 0.02				
Medium	≥ 0.3	≥ 0.13				
Large	≥ 0.5	≥ 0.26				

BSFS: Bristol Stool Form Scale; BMI: body mass index; NRS: numerical rating scale; SBM: spontaneous bowel movement; Sx: symptom; SS: subscale.

## Discussion

The study revealed that approximately three-quarters of initial non-responders at 2 weeks remained non-responders at 4 weeks. Notably, early therapeutic response at 2 weeks was the strongest independent predictor of subsequent outcomes at 4 weeks, as confirmed by multivariate analysis. These results suggest that early treatment modification may benefit the management of patients with treatment-resistant chronic constipation.

To better contextualize these findings, it is important to consider the role of patient-reported outcomes (PROs) in chronic constipation management. The Food and Drug Administration (FDA) advocates the use of PROs for conditions where symptom relief is a primary goal [13]. Although several PRO tools exist for diagnosing and assessing chronic constipation [14-18], validated instruments tailored to Japanese patients have been lacking until recently. The newly validated CC-TEST serves as a comprehensive PRO tool, evaluating symptom severity, defecation status, impact on daily life, and therapeutic efficacy [10].

The CC-TEST assesses therapeutic response through patient’s impression, NRS, and SBM frequency. While no single definition optimally captures treatment response in chronic constipation, global binary endpoints are recommended to detect minimal yet clinically meaningful changes. The NRS, originally developed for chronic pain and recommended by the FDA for abdominal pain in irritable bowel syndrome [10, 19, 20], offers higher compliance, responsiveness, and applicability than visual analog scales. SBM frequency is also widely accepted as a key outcome measure in chronic constipation studies. Our results demonstrated both commonalities and distinctions in factor associations across these three definitions, underscoring the multifaceted nature of treatment response assessment.

Despite the availability of various treatments, satisfaction rates with OTCM and prescribed therapies for chronic constipation remain below 50% [21-24], likely reflecting limited efficacy and adverse effects [25]. Current treatment guidelines recommend lifestyle modifications and osmotic laxatives such as magnesium salts and PEG as first-line therapies [6, 7], followed by sequential use of laxatives, newer drug classes, or surgical interventions for initial non-responders [6, 7, 26-28]. Although BSFS and SBM frequency are effective measures to assess treatment response [7], predicting which patients will respond to initial treatment remains challenging. Our findings suggest that incorporating assessment of therapeutic response at 2 weeks could improve prediction of 4-week outcomes, informing timely treatment adjustments.

The terminology surrounding treatment-resistant constipation remains inconsistent. The term “refractory” typically refers to insufficient response to drugs [29]. Given the chronic nature of constipation, treatment evaluation over at least 4 weeks has been considered necessary to balance efficacy and safety [30]. However, this delay may prolong patient suffering. Previous clinical trials have observed plateau effects in symptom improvement at 2 and 4 weeks with several drugs [31-33]. Taken together with our results, these data support the clinical practice of considering treatment modification as early as 2 weeks for patients who show poor initial response.

Nonetheless, approximately one-quarter of the patients who did not respond at 2 weeks became responders at 4 weeks in the present study. These delayed responses suggest that continuing the same medication may still be beneficial for a subset of patients. Such improvement might be attributed to gradual pharmacologic onset, cumulative effects, or enhanced adherence over time. Therefore, early treatment modification at 2 weeks should be considered selectively, through shared decision-making with patients. Further large-scale prospective studies are warranted to identify the specific factors that indicate which patients may benefit from early treatment modification.

Several limitations of the present study must be acknowledged. First, the study did not reach the initially planned sample size because of COVID-19-related restrictions, which may have introduced selection bias and potentially limited the generalizability of the findings. Although the final sample size of 97 patients was sufficient to detect the observed effect sizes, the reduction may have limited the statistical power to identify smaller but potentially meaningful associations. Second, the study allowed use of various constipation medications approved in Japan without standardizing treatment types, making it difficult to isolate the effects of individual drugs on therapeutic efficacy. Third, because the data primarily represent patients with moderate to severe constipation, our findings may not be generalizable to those with milder symptoms or different demographic profiles. Fourth, despite efforts to minimize information bias by anonymizing data and blinding assessors, PROs inherently carry some degree of subjective bias. Fifth, although key patient characteristics were recorded and adjusted for when possible, residual confounding from unmeasured factors cannot be ruled out. Finally, because the number of patients enrolled per center was small, adjusting for center-level effects using a mixed-effects model was statistically unstable and therefore not feasible. Although unmeasured site-specific factors may have influenced the results, treatment response in chronic constipation likely depends mainly on individual-level characteristics, and center-level differences are expected to have had minimal impact. Despite these limitations, the treatment regimens used in this study closely reflect real-world clinical practice in Japan, thereby enhancing the clinical relevance of our findings. Future research with larger sample sizes and more controlled medication protocols is warranted to validate these findings in broader populations.

In conclusion, the present study demonstrated that approximately three-quarters of patients who did not respond to initial treatment at 2 weeks remained non-responders at 4 weeks. Therefore, for patients with chronic constipation who show a poor initial response, early modification of the medication regimen at 2 weeks - rather than continuing the original treatment for 4 weeks - appears to be a reasonable therapeutic strategy when guided by shared decision-making with patients. Such an approach may help improve patients’ QOL.

## Supplementary Material

Suppl 1 Prescribed constipation medications in treatment efficacy study.

## Data Availability

The data that support the findings of this study are available from the corresponding author upon reasonable request. The data are not publicly available due to privacy or ethical restrictions.

## Abbreviations

BSFS: Bristol Stool Form Scale
CC-TEST: Chronic Constipation-Therapeutic Efficacy and Satisfaction Test
COVID-19: coronavirus disease 2019
FDA: Food and Drug Administration
NRS: numeric rating scale
OTCM: over-the-counter medication
PEG: polyethylene glycol
PRO: patient-reported outcome
QOL: quality of life
SBM: spontaneous bowel movement
SS: subscale
Sx: symptom
