# Supplementary material for: Early Therapeutic Response Predicts Outcome in Chronic Constipation: A Multicenter Prospective Observational Study
Source: Gastroenterology Res. 2026 Jan 4;19(1):43–53. doi: 10.14740/gr2071 (PMC12978407; doi:10.14740/gr2071)
Supplement: Suppl 1 — Prescribed constipation medications in treatment efficacy study. [file gr-19-01-043-s001.docx]

**Suppl 1.** Prescribed constipation medications in treatment efficacy study

|  | **Number of patients** |
| --- | --- |
| Prescribed constipation medications |  |
| New constipation remedy | 87 |
| Macrogol 4000 | 52 |
| Elobixibat | 21 |
| Linaclotide | 8 |
| Lubiprostone | 6 |
| Lactulose | 7 |
| Osmotic laxatives (MgO) | 23 |
| Stimulant laxatives | 20 |
| Kampo medicine | 7 |
| Probiotics | 15 |
| Other drugs | 3 |
| Number of prescription laxatives |  |
| 1 | 55 |
| 2 | 22 |
| 3 | 14 |
| 4 | 3 |
| 5 | 3 |
